# ¿Salud para quién? Interseccionalidad y sesgos de la inteligencia artificial para el diagnóstico clínico

**DOI:** 10.23938/ASSN.1077

**Published:** 2024-07-04

**Authors:** Sua Amaya-Santos, Jaime Jiménez-Pernett, Clara Bermúdez-Tamayo

**Affiliations:** 1 Escuela Andaluza de Salud Pública Granada España; 2 Jagiellonian University of Krakow Cracovia Polonia; 3 Universidad de Granada Granada España; 4 Instituto de Investigación Biosanitaria ibs.GRANADA Granada España; 5 CIBER de Epidemiología y Salud Pública (CIBERESP) España

Las tecnologías basadas en Inteligencia Artificial (IA) han experimentado en los últimos años un crecimiento significativo en el ámbito de la sanidad, con la promesa de mejorar tanto la atención médica como la salud pública^1^. Así, el potencial evidenciado durante la pandemia por COVID-19 hace que se constituyan como elementos centrales en las estrategias para el desarrollo de sistemas de información sanitaria^2^.

Las aplicaciones de la IA se extienden a todo el ciclo de atención al paciente, destacando su habilidad para apoyar el diagnóstico gracias a su capacidad para analizar imágenes médicas como radiografías, resonancias magnéticas y tomografías computarizadas, a través de la identificación de patrones y anomalías que incluso podrían escapar a la detección humana^3^.

El uso de estas tecnologías podría conllevar a diagnósticos más tempranos y precisos, así como a minimizar los errores, como han demostrado ya algunas investigaciones en radiología, patología y dermatología que han revelado una mayor precisión diagnóstica al comparar los resultados obtenidos mediante IA con los realizados por profesionales^4^. No obstante, el optimismo hacia los beneficios potenciales de la IA, o *tecno-optimismo*, podría obviar el riesgo de que se perpetúen, exacerben o profundicen los prejuicios y las disparidades en la atención sanitaria por cuestiones de género, raciales o étnicas produciendo inferencias sesgadas. La salud está influida por vínculos complejos entre factores biológicos, socioeconómicos y contextuales, los cuales a menudo se encuentran rodeados de variables de confusión, como el estigma y los estereotipos, que pueden llevar a la representación errónea de los datos^5^, y a sesgos cognitivos^6^. Las investigaciones han revelado que los mecanismos de la IA pueden amplificar los comportamientos discriminatorios que son representativos de desigualdades arraigadas^7^.

## INTERSECCIONALIDAD EN SALUD

La interseccionalidad constituye un marco esencial para analizar cómo factores como la raza, el género y otras identidades sociales se entrelazan y se combinan, generando manifestaciones de opresión y desigualdad. La idea subyacente es que la investigación científica y la práctica clínica se ha centrado en los miembros más privilegiados, *socavando* los esfuerzos por implementar iniciativas antidiscriminatorias^8^.

La evidencia indica que, ante características de las personas, el personal sanitario puede mostrar prejuicios inconscientes que influyen en su interpretación de los síntomas, los resultados de las pruebas diagnósticas y las recomendaciones de tratamiento^8^. Denominamos *sesgo interseccional* a la discriminación de manera sistemática e injusta contra ciertos individuos o grupos en beneficio de otros, y que puede manifestarse como un sesgo cognitivo, lo que afecta a los procesos de toma de decisiones y dar lugar a disparidades. Es decir, puede conducir a errores diagnósticos, tratamientos subóptimos y daños a los pacientes^9^. Este sesgo podría empeorar la marginación de las minorías y ampliar la brecha de las desigualdades en salud.

**Figura 1:**
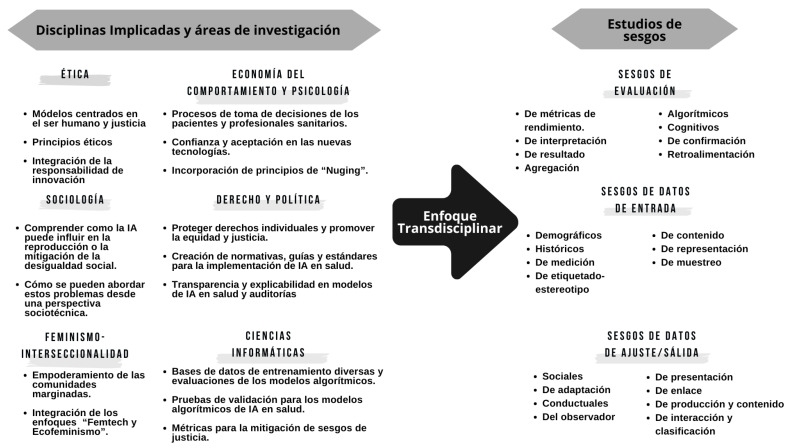
Estudio de sesgos para su mitigación con enfoque transdisciplinar

Las IA pueden reflejar y amplificar los sesgos presentes en los datos que utilizan, lo que podría perpetuar la discriminación. Este fenómeno se ha abordado desde diversas disciplinas para mitigarlo, dando lugar a diferentes propuestas de clasificación de sesgos. En la figura 1 se describen las disciplinas y los sesgos más frecuentes relacionados con la entrada de datos, algoritmos, evaluación y ajuste/salida^10^^,^^11^.

## EVIDENCIAS SOBRE EL SESGO DE INTERSECCIONALIDAD EN LA INTELIGENCIA ARTIFICIAL SANITARIA

Algunas tecnologías de IA han demostrado ser discriminatorias en función de características como el sexo/género, siendo las mujeres generalmente las más afectadas^12^. Un estudio reciente describe cómo robots entrenados con grandes conjuntos de datos exhibían un comportamiento estereotipado y sesgado en términos de género y raza^13^. Otro estudio mostró sesgo de interseccionalidad del proveedor, documentando los síntomas de pacientes afroamericanos a partir de registros médicos de manera peyorativa^14^. Se ha demostrado que, a partir de imágenes radiológicas, las redes neuronales convolucionales (CNN) pueden subdiagnosticar erróneamente a grupos vulnerables (en particular, hispanos y pacientes con *Medicaid* en Estados Unidos) en una proporción mayor a los pacientes blancos^15^. También se ha evidenciado que un sistema de IA entrenado y validado únicamente en personas con fácil acceso a los servicios produce un sesgo en el diagnóstico a minorías con bajo acceso a la atención sanitaria^14^. Un último caso se refiere al desarrollo de calculadoras de evaluación del riesgo de fractura ósea. Estas calculadoras realizaron correcciones por país para tener en cuenta las diferentes incidencias de osteoporosis (por ejemplo, el riesgo se ajustó a la baja para las mujeres de raza negra con incidencia notificada), pero estas correcciones también generaron infradiagnóstico que sería parte del conjunto de datos que se utilizan para el entrenamiento de IA^16^. Suele asumirse que un aumento en la diversidad cambiará la manera en que se entrenan las IA y, por ende, sus predicciones, haciéndola más inclusiva y reduciendo sus riesgos. Sin embargo, esta suposición aún no ha sido probada^9^, y lo cierto es que la minimización del sesgo requiere acciones más complejas.

**Figura 2:**
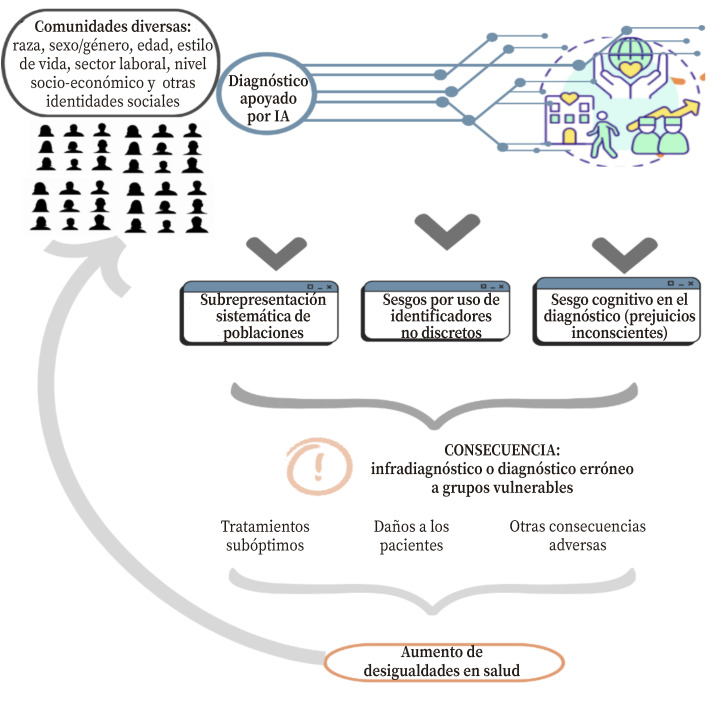
Sesgo de interseccionalidad en el diagnóstico apoyado por Inteligencia Artificial.

En la Figura 2 se esquematiza la interacción de los diferentes mecanismos, y cómo los sesgos de interseccionalidad pueden surgir en el contexto de la IA y sus implicaciones. En primer lugar, la falta de diversidad en los conjuntos de datos digitales usados por los algoritmos de IA puede amplificar la subrepresentación sistemática de ciertas poblaciones^9^. En segundo lugar, los marcadores de identidad pueden ocasionar malentendidos culturales por no recoger la complejidad de los fenómenos de la interseccionalidad (como sexo/género, edad, estilo de vida, o sector laboral) de manera conjunta) estatificándolas y clasificarlos de manera discreta^17^ y la asunción de un estatus de hecho imparcial erróneo. Finalmente, hay que considerar el sesgo cognitivo de los profesionales (relacionados con su percepción y razonamiento) implícito en los conjuntos de datos. La toma de decisiones clínicas parece ser el resultado de dos modos distintos de procesamiento cognitivo^18^: el proceso consciente de evaluar opciones basadas en una combinación de utilidad, riesgo, capacidades y/o influencias sociales, o sistema tipo 2, y la cognición automática o sistema tipo 1, referente a los procesos en gran parte inconscientes que ocurren en respuesta a señales ambientales o emotivas y basados en heurísticos arraigados, previamente aprendidos^11^^,^^19^.

El sesgo de interseccionalidad puede conllevar al infradiagnóstico o diagnóstico erróneo de grupos vulnerables, lo que a su vez podría conllevar a tratamientos subóptimos, daños a los pacientes, amplificar las disparidades de salud y perpetuar la marginalización de grupos desatendidos. Para reconocer y resolver estos problemas es crucial medir el sesgo, tanto en los modelos finales como en los conjuntos de datos, lo que ha llevado al desarrollo de métricas para la detección de sesgos en los últimos años. Así, un estudio empleó diferentes taxonomías de métricas de sesgo demográfico para detectar el sesgo representacional y estereotípico en bases de datos para el entrenamiento de una IA para el reconocimiento de expresiones faciales^20^.

## POLÍTICAS, MARCOS Y DIRECTRICES EN MATERIA DE IA

La normativa desempeña un papel central en el establecimiento de un marco defensivo frente a las amenazas percibidas, anticipadas y reales de la IA^21^. Los esfuerzos para abordar sus riesgos e implicaciones sociales y éticas han dado lugar a un *corpus* documental cada vez más extenso, dado que la mayoría de los instrumentos regulatorios existentes no fueron redactados teniendo en cuenta la magnitud de los cambios de la IA^22^. Distintos países avanzan en el abordaje de estas brechas. Por ejemplo, la Comisión Europea propuso en 2021 una Ley de Inteligencia Artificial, actualmente en desarrollo, aplicable a los sistemas de IA en salud, aunque por ahora no aborda suficientemente las especificidades de este campo^23^.

**Figura 3:**
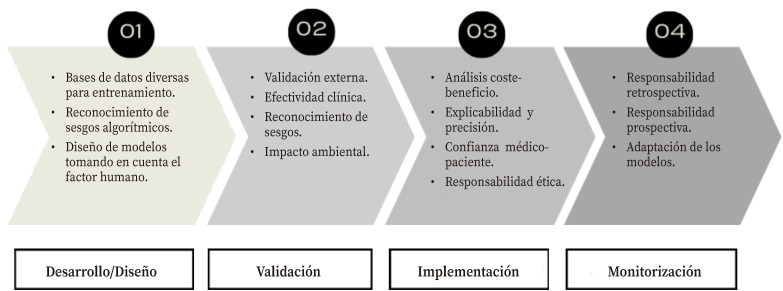
Proceso para una Inteligencia Artificial responsable en salud. Elaboración propia a partir de Sujan y col 2023^29^.

Otras iniciativas se refieren a retos en materia de responsabilidad que requieren una atención política urgente, como derechos humanos, cuestiones sociales, económicas y medioambientales, y valores democráticos, integrados bajo el nombre de *Inteligencia artificial responsable* (IAR)^24^ (Fig. 3). Iniciativas tales como los *Lineamientos para la Inteligencia Artificial Responsable* o la herramienta de evaluación *Responsabilidad de las soluciones digitales en salud* impulsadas recientemente por compañías de *Big Tech*, el mundo académico e institutos de investigación, junto con gobiernos y ONG. Desafortunadamente, hasta ahora ha tenido escaso impacto en la práctica real de la IA^25^.

## DESAFÍOS PARA EL FUTURO

Abordar la cuestión de *¿Salud para quién?* requiere ir más allá de directrices e intenciones, con normativas que prioricen el desarrollo y el uso responsable de la IA centrándose en el bienestar de todos los individuos, para que los responsables políticos refuercen su papel en un campo tan dinámico.

La confiabilidad de la IA es un cuello de botella crítico en su adopción. El fenómeno de la *caja negra* es una crítica a la mayoría de la IA actual, ya que carece de transparencia, de explicación, y sus resultados no pueden generalizarse^26^. Una sugerencia general es aumentar la diversidad entre el personal investigador de distintas disciplinas que trabajan con macrodatos/IA y equidad. Los sesgos discriminatorios se pueden prevenir mediante la incorporación de una amplia gama de perspectivas, ya que esto puede reducir la probabilidad de generar sesgos basados en puntos de vista singulares^27^.

Se necesita más investigación para determinar la mejor manera de detectar el sesgo relacionado con la interseccionalidad por el uso de identificadores no discretos^16^. Esto implica que las políticas nacionales, las instituciones y las comunidades de investigación tendrían que profundizar en el desarrollo de estándares armonizados en interseccionalidad e identidades sociales^9^^,^^17^.

Los marcos legales existentes tienden a poner un énfasis en la seguridad física y la privacidad, descuidando factores igualmente importantes como la diversidad, la subrepresentación sistemática de poblaciones y la influencia del sesgo cognitivo implícito en los datos^28^. Es necesario, además, considerar tanto la responsabilidad retrospectiva como la responsabilidad prospectiva. La primera implica rendir cuentas y/o la necesidad de poder comprender y explicar las decisiones de dichos sistemas. Por otra parte, la responsabilidad prospectiva exige que todas las partes interesadas asuman el deber de garantizar un despliegue ético de la IA^29^.
